# Comment on: “Dexmedetomidine as a useful adjunctive agent for preventing lung injury during emergence from anesthesia and tracheal extubation”—a reply

**DOI:** 10.1186/s40981-025-00770-3

**Published:** 2025-02-01

**Authors:** Hayato Arime, Takashi Asai

**Affiliations:** Department of Anesthesiology, Dokkyo Medical University Saitama Medical Center, Koshigaya, Saitama 343-8555 Japan

To the editor

We thank Dr. Watanabe for his interest in our case report [1]. We fully agree with his statement that there is no established method to minimize the risk of lung injury during emergence from anesthesia and tracheal extubation. We have suggested that one effective method is to insert a supraglottic airway while the double-lumen tube is in place, to remove the tube under deep anesthesia, leaving the supraglottic airway until the patient can respond to verbal command [1]. The usefulness of this method is confirmed by Dr. Watanabe’s case, in which he used the same method under continuous infusion of dexmedetomidine.

Some drugs, such as fentanyl, remifentanil, lidocaine, dexmedetomidine, have been reported to be effective in preventing responses to stimuli associated with removal of a tracheal tube [2–5]. In our case, no such drugs were used during emergence from anesthesia [1], because respiratory complications are unlikely to occur when a supraglottic airway is removed after the patient has gained consciousness [6, 7]. Although we acknowledge that Dr. Watanabe provided dexmedetomidine mainly to prevent delirium, and not respiratory complications, any sedative effect might increase the risk of another type of respiratory complications, such as upper airway obstruction or respiratory depression.

Here, we point out another use of a supraglottic airway in a patient with a double-lumen tube in place. In some patients with cardiorespiratory complications, mechanical ventilation may be required postoperatively. In such a case, a double-lumen tube needs to be exchanged by a single-lumen tracheal tube, but there is a risk of difficulty in inserting a single-lumen tracheal tube. We have been using a supraglottic airway for a smooth exchange of tubes, with the following methods. After confirming sufficiently deep anesthesia and neuromuscular blockade, a supraglottic airway is inserted while a double-lumen tube is still in place. A fiberoptic bronchoscope is passed through a single-lumen tracheal tube, and the combination was passed through a supraglottic airway. The fiberscope is passed through the vocal cords into the trachea, alongside the double-lumen tube. The double-lumen tube is removed, and a single-lumen tube is then passed over the fiberscope into the trachea.

We have been using this method in patients who required postoperative mechanical ventilation (such as those underwent open esophagectomy or video-assisted thoracoscopic surgery), and exchange of tubes was always successful with a duration of apnea less than 15 s. We have reported the same method for exchange of single-lumen tubes (oral to oral [8] and nasal to oral [9]).

In conclusion, as Dr. Watanabe points out, we believe that, in patients with cardiopulmonary complications, the use of a supraglottic airway after surgery is potentially useful in minimizing respiratory complications associated with removal of a double-lumen tube.

## Data Availability

The data in this letter are available from the corresponding author on reasonable requests.
